# 

**Published:** 1872-01-15

**Authors:** 


### Medical Examiner.

A Semi-Monthly Journal of Medical Sciences.

No. 2. CHICAGO, JANUARY 15, 1872. Vol. XIII.

TERMS.—Yearly Subscriptions, Three Dollars, in advance. Single Numbers Fifteen Cents.
All Communications should be addressed to Publishers Medical Examiner, 797 Wabash Av., Chicago.

